# Genome Sequences of Rotavirus A Strains Ty-1 and Ty-3, Isolated from Turkeys in Ireland in 1979

**DOI:** 10.1128/genomeA.01565-15

**Published:** 2016-01-14

**Authors:** Yuji Fujii, Hiromichi Mitake, Daigo Yamada, Makoto Nagai, Kota Okadera, Naoto Ito, Kazuma Okada, Kento Nakagawa, Tetsuya Mizutani, Makoto Sugiyama

**Affiliations:** a Laboratory of Zoonotic Diseases, Faculty of Applied Biological Sciences, Gifu University, Yanagido, Gifu, Japan; b The United Graduate School of Veterinary Sciences, Gifu University, Yanagido, Gifu, Japan; c Research and Education Center for Prevention of Global Infectious Disease of Animal, Tokyo University of Agriculture and Technology, Saiwaimachi, Fuchu, Tokyo, Japan

## Abstract

To obtain complete genome sequences of turkey rotavirus A strains Ty-1 and Ty-3, we sequenced the gene segments that had not been decoded previously. The genotype constellations of the respective strains were determined to be G17-P[38]-I4-R4-C4-M4-A16-N4-T4-E4-H4 and G7-P[35]-I4-R4-C4-M4-A16-N4-T4-E11-H14. Notably, their VP4 and NSP5 genes were classified into novel genotypes.

## GENOME ANNOUNCEMENT

Rotavirus A (RVA) is one of the major causes of diarrhea in infants and young animals (1). The genome consists of 11 segments of double-stranded RNA that encode six structural proteins (VP1-4, VP6, and VP7) and five or six nonstructural proteins (NSP1-5/6) (1). The genotypes of VP7-VP4-VP6-VP1-VP2-VP3-NSP1-NSP2-NSP3-NSP4-NSP5 genes are determined by a classification system proposed by the Rotavirus Classification Working Group (RCWG) (2) and are indicated as Gx-P[x]-Ix-Rx-Cx-Mx-Ax-Nx-Tx-Ex-Hx, where x represents the number of genotypes (3).

To understand the ecology and evolution of RVA, it is necessary to accumulate genetic information on RVA strains circulating in various host species. However, while numerous human and animal RVAs have been genetically analyzed at the whole-genome level, avian RVAs have not been extensively studied: currently, complete genome sequences of only six avian RVA strains have been determined (references 4–7 and unpublished data).

Avian RVA strains Ty-1 and Ty-3 were isolated from turkeys in Ireland in 1979 (8) and have been considered as representative avian RVAs. However, their complete genome sequences have not been determined yet: only the VP6-encoding segment of both strains and the NSP2- and VP7-encoding segments of the Ty-1 strain were previously sequenced (9–11). To obtain complete genome sequences of Ty-1 and Ty-3, we determined the gene segments that had not been decoded previously.

Viral RNA was extracted using ISOGEN-LS (Nippon gene). Libraries for next-generation sequencing (NGS) were constructed using the NEBNext Ultra RNA library prep kit for Illumina version 2.0 (New England Biolabs) according to the manufacturer’s guidelines. NGS was carried out on the MiSeq bench-top sequencer (Illumina). Contigs were assembled from the obtained sequence using the *de novo* assembly command with default parameters in CLC Genomics Workbench 6.0 (CLC bio). The nucleotide sequences of 5′ and 3′ untranslated regions (UTRs) were determined by a 3′ rapid amplification of the cDNA end method (3′ RACE) (4). The genotypes were determined according to the guidelines of the RCWG (3) using the online genotyping tool RotaC (http://rotac.regatools.be) (12) and BLAST (http://blast.ncbi.nlm.nih.gov/Blast.cgi).

Genotyping by RotaC revealed that all viral genes except for the VP4 gene of Ty-1 and NSP5 gene of Ty-3 were classified into previously established avian RVA genotypes. Meanwhile, the Ty-1 VP4 gene and Ty-3 NSP5 gene were not closely related to the respective genes of any known RVA strains: they showed the highest nucleotide identities of 77.4% with the VP4 gene of velvet scoter RVA strain RK1 (13) and of 83.9% with the NSP5 gene of pigeon RVA strain PO-13, respectively, which are clearly lower than the cut-off values defined by RCWG for genotyping (80% for VP4 gene and 91% for NSP5 gene) (3). RCWG officially confirmed that the Ty-1 VP4 gene and Ty-3 NSP5 gene were classified into new genotypes, P[38] and H14, respectively. Taken together, the genotype constellations of Ty-1 and Ty-3 were determined as G17-P[38]-I4-R4-C4-M4-A16-N4-T4-E4-H4 and G7-P[35]-I4-R4-C4-M4-A16-N4-T4-E11-H14, respectively.

### Nucleotide sequence accession numbers.

The nucleotide sequences determined in this study have been deposited in GenBank under the accession numbers LC088107 to LC088124.
